# Photocatalytic Degradation of Methylene Blue and Anticancer Response of In_2_O_3_/RGO Nanocomposites Prepared by a Microwave-Assisted Hydrothermal Synthesis Process

**DOI:** 10.3390/molecules28135153

**Published:** 2023-06-30

**Authors:** ZabnAllah M. Alaizeri, Hisham A. Alhadlaq, Saad Aldawood, Mohd Javed Akhtar, Aziz A. Aziz, Maqusood Ahamed

**Affiliations:** Department of Physics and Astronomy, College of Science, King Saud University, Riyadh 11451, Saudi Arabia

**Keywords:** In_2_O_3_/RGO, microwave hydrothermal method, degradation, cytotoxicity effect, biocompatibility

## Abstract

The incorporation of graphene with metal oxide has been widely explored in various fields, including energy storage devices, optical applications, biomedical applications, and water remediation. This research aimed to assess the impact of reduced graphene oxide (RGO) doping on the photocatalytic and anticancer properties of In_2_O_3_ nanoparticles. Pure and In_2_O_3_/RGO nanocomposites were effectively synthesized using the single-step microwave hydrothermal process. XRD, TEM, SEM, EDX, XPS, Raman, UV–Vis, and PL spectroscopy were carefully utilized to characterize the prepared samples. XRD data showed that synthesized In_2_O_3_ nanoparticles had high crystallinity with a decreased crystal size after RGO doping. TEM and SEM images revealed that the In_2_O_3_ NPs were spherical and uniformly embedded onto the surface of RGO sheets. Elemental analysis of In_2_O_3_/RGO NC confirmed the presence of In, O, and C without impurities. Raman analysis indicated the successful fabrication of In_2_O_3_ onto the RGO surface. Uv–Vis analysis showed that the band gap energy was changed with RGO addition. Raman spectra confirmed that In_2_O_3_ nanoparticles were successfully anchored onto the RGO sheet. PL results indicated that the prepared In_2_O_3_/RGO NCs can be applied to enhance photocatalytic activity and biomedical applications. In the degradation experiment, In_2_O_3_/RGO NCs exhibited superior photocatalytic activity compared to that of pure In_2_O_3_. The degradation efficiency of In_2_O_3_/RGO NCs for MB dye was up to 90%. Biological data revealed that the cytotoxicity effect of In_2_O_3_/RGO NCs was higher than In_2_O_3_ NPs in human colorectal (HCT116) and liver (HepG2) cancer cells. Importantly, the In_2_O_3_/RGO NCs exhibited better biocompatibility against human normal peripheral blood mononuclear cells (PBMCs). All the results suggest that RGO addition improves the photocatalytic and anticancer activity of In_2_O_3_ NPs. This study highlights the potential of In_2_O_3_/RGO NCs as an efficient photocatalyst and therapeutic material for water remediation and biomedicine.

## 1. Introduction

Nanocomposites (NCs) have shown promising potential in medical applications, including antibacterial, cancer diagnostics, and therapy [1,2,3,4]. Among these nanocomposites (NCs), nanomaterials based on metal oxide nanoparticles (NPs) are attracting interest in a number of areas of science and technology due to their desirable properties, such as being low-cost, non-toxic, renewable, and excellent semiconductors [5,6]. Furthermore, graphene is a sp^2^-bonded hexagonal network of carbon atoms [7]. It has excellent properties such as a high specific surface area, favorable electron transportation, tunable band gap, good thermal and electrical conductivity, and biocompatibility [8,9]. On the other hand, graphene oxide (GO) and reduced oxide (RGO) have attracted significant attention due to their unique properties, such as high surface area, good electrical conductivity, and excellent chemical stability [10,11].

Several studies have investigated the photocatalytic degradation of methylene blue using metal oxide nanoparticles (NPs). For instance, [12] reported a significant degradation of MB under visible light irradiation using TiO_2_ NPs. Similarly, other metal oxide nanoparticles, such as ZnO NPs have also exhibited promising photocatalytic properties for MB degradation [13]. The cytotoxicity of metal oxide NPs towards various cancer cell lines, including breast, lung, prostate, and liver cancer have been reported in numerous studies. For example, Kokila et al. [14] showed that silver oxide (Ag_2_O) NPs exhibited strong anti-cancer effects on lung cancer cells.

The incorporation of metal oxide NPs into graphene has been achieved through various approaches to synthesize novel nanocomposites (NCs), which solve certain challenges associated with pure metal oxide NPs [15,16]. For example, ZnO/RGO NCs [17], ZnO-MoS_2_/RGO NCs [18], MoS_2_-Fe_2_O_3_/RGO NCs [19], Ag/ZnO/RGO NCs [20], and Ce/ZnO/RGO NCs [21] have been successfully prepared and have potential in biomedicine and environmental remediation applications. Different approaches including chemical, physical, and biological methods have been used to synthesize metal oxide NPs with RGO as NCs [22,23,24]. Bibi et al. [25] reported the synthesis of Fe_2_O_3_-TiO_2_/RGO NCs through a hydrothermal approach, which demonstrated remarkable methyl blue degradation capabilities due to the synergistic effect between Fe_2_O_3_-TiO_2_NPs and RGO. Indium oxide nanoparticles (In_2_O_3_ NPs) based on graphene have been extensively studied for potential applications such as high-performance supercapacitors [26,27], gas sensing [28], energy storage devices [29], biomedicine [30], and water remediation [31]. For example, Devi and Singh [32] reported that doping RGO in In_2_O_3_ plays a role in improving the photocatalytic performance due to its surface area and improved charge separation.

This study aimed to enhance the properties of In_2_O_3_ NPs by RGO doping on their photocatalytic and anticancer performance. XRD, SEM, TEM, EDX, XPS, Raman scattering, UV–Vis, and PL spectroscopy were used to characterize the synthesized samples. The results show that In_2_O_3_/RGO NCs exhibited superior photocatalytic activity compared to pure In_2_O_3_ NPs. Cytotoxicity assessment demonstrated that In_2_O_3_/RGO NCs displayed increased cytotoxicity against human colorectal (HCT116) and liver (HepG2) cancer cells compared to In_2_O_3_ NPs. These results indicate that In_2_O_3_/RGO NCs exhibit improved photocatalytic and anticancer properties.

## 2. Results and Discussion

### 2.1. Structural Study

**Figure 1:**
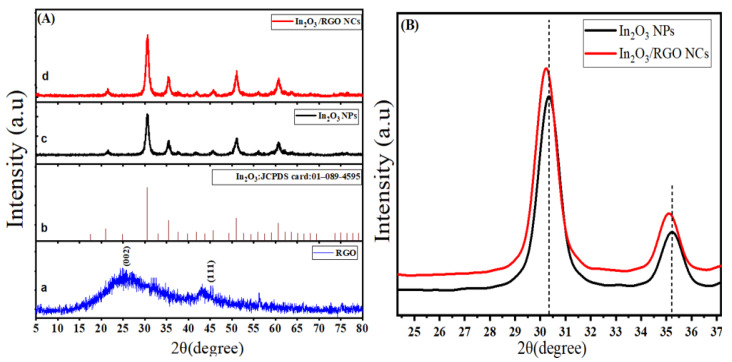
XRD Spectra of RGO (**a**), the standard pattern of monoclinic In_2_O_3_ (JCPDS card: 01-089-4595) (**b**), In_2_O_3_ NPs (**c**), and In_2_O_3_/RGO NCs (**d**) (**A**) and high-resolution XRD spectra of In_2_O_3_ NPs, and In_2_O_3_/RGO NCs (**B**).

The crystal structure and phase composition of prepared samples were analyzed by X-ray diffraction (XRD). Figure 1A shows XRD spectra of RGO, pure In_2_O_3_ NPs, and In_2_O_3_/RGO NCs. As depicted in Figure 1Aa, XRD spectra of RGO demonstrate a characteristic peak of graphene at 26.1° to (002) plane [33]. It can be seen in Figure 1Ac that the main diffraction peaks of pure In_2_O_3_ NPs are assigned at 30.3°, 35.2°, 51.7°, and 58.3° indexed to the (222), (400), (440), and (622) planes of cubic In_2_O_3_, respectively. However, the peaks of XRD spectra of pure In_2_O_3_ NPs were matched with the standard diffraction pattern (Figure 1Ac) of In_2_O_3_ (JCPDS card No:01–089-4595). XRD spectra of In_2_O_3_/RGO NCs (Figure 1Ad) showed that the diffraction peaks at 30.1°, 35°, 51.2°, and 58.2° can be indexed to the ((222), (400), (440), and (622) planes. This phenomenon indicates that they were slightly shifting to a low angle (Figure 1B). Results indicated that In_2_O_3_/RGO NCs were further crystallized due to their high peak intensity and sharp peak width, which were matched with earlier studies [34,35,36]. The average crystal sizes of pure In_2_O_3_ NPs and In_2_O_3_/RGO NCs were 17 nm and 13 nm (estimated by the Debye Scherrer formula), respectively. It was observed that the average crystal sizes were decreased with RGO doping. XRD results show that the In_2_O_3_ NPs were anchored on the surface of RGO due to the change in the intensities of peaks [28]. Obtained results agree with previous studies [36,37].

### 2.2. Morphological Study

**Figure 2:**
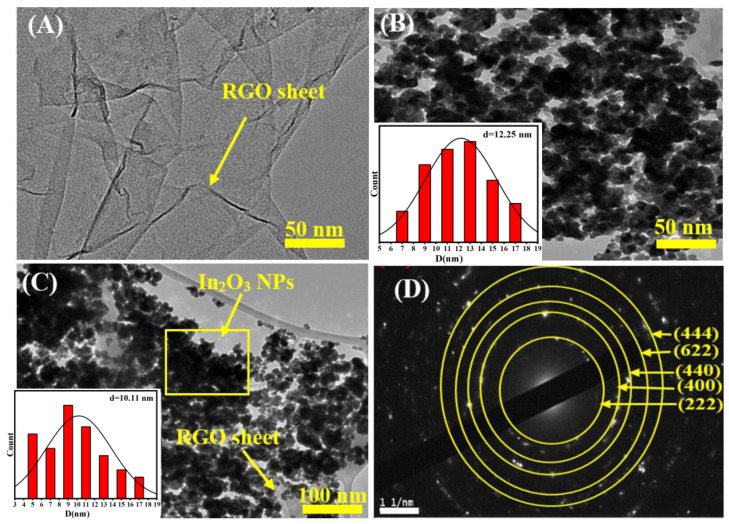
TEM image of RGO (**A**), TEM image of pure In_2_O_3_ NPs (**B**), TEM image of In_2_O_3_/RGO NCs (**C**), and selected area electron diffraction (SAED) pattern of In_2_O_3_/RGO NCs (**D**).

TEM analysis was further applied to characterize the morphologies and particle sizes of prepared nanocomposites (NCs). Figure 2A–D presents the TEM images and a selected area electron diffraction (SEAD) pattern of RGO, pure In_2_O_3_ NPs, and In_2_O_3_/RGO NCs. It can be seen in Figure 2A that the TEM image of RGO was shown as thin and transparent sheets with wrinkles and folds. Thus, the thickness of the RGO sheets cannot be accurately determined from the TEM image [38]. We observe that the TEM image (Figure 2B) of pure In_2_O_3_ NPs was uniformly distributed spherically as found in an earlier investigation [39]. As shown in Figure 2C, the In_2_O_3_ NPs were anchored onto the surface of the RGO sheets with a homogeneous dispersion owing to the interaction between RGO and In_2_O_3_ NPs [23,24,32,40]. Table 1 shows the average particle sizes of In_2_O_3_NPs and In_2_O_3_/RGO NCs, which were 12 nm and 10 nm, respectively. The crystal structure and orientation of the In_2_O_3_/RGO NCs are shown in the SEAD pattern (Figure 2D). However, the SEAD pattern shows a series of diffraction rings and spots, which correspond to the planes and directions of the In_2_O_3_ and RGO crystals. The diffraction spots are indexed to the (222), (400), (440), (622), and (444) planes of In_2_O_3_, which agree with the standard JCPDS data (No. 01–089-4595). TEM results revealed that the In_2_O_3_ NPs were successfully attached to the surface of the RGO sheet as novel In_2_O_3_/RGO NCs.

**Figure 3:**
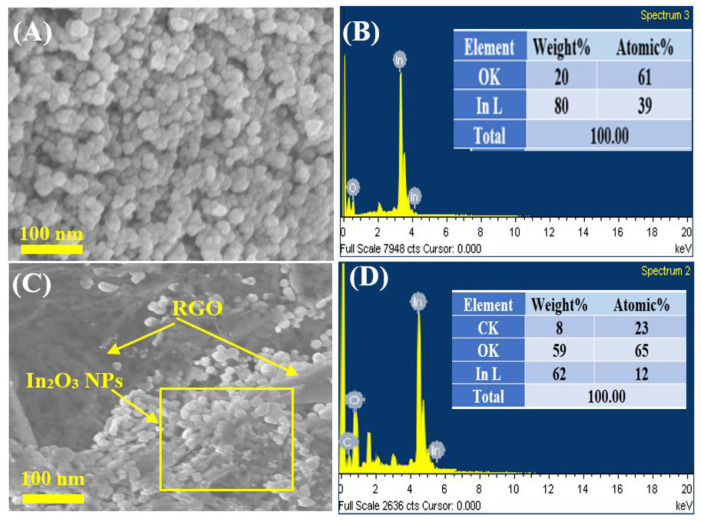
SEM image of pure In_2_O_3_ NPs (**A**), EDX spectra of pure In_2_O_3_ NPs (**B**), SEM image of In_2_O_3_/RGO NCs (**C**), and EDX spectra of In_2_O_3_/RGO NCs (**D**).

**Figure 4:**
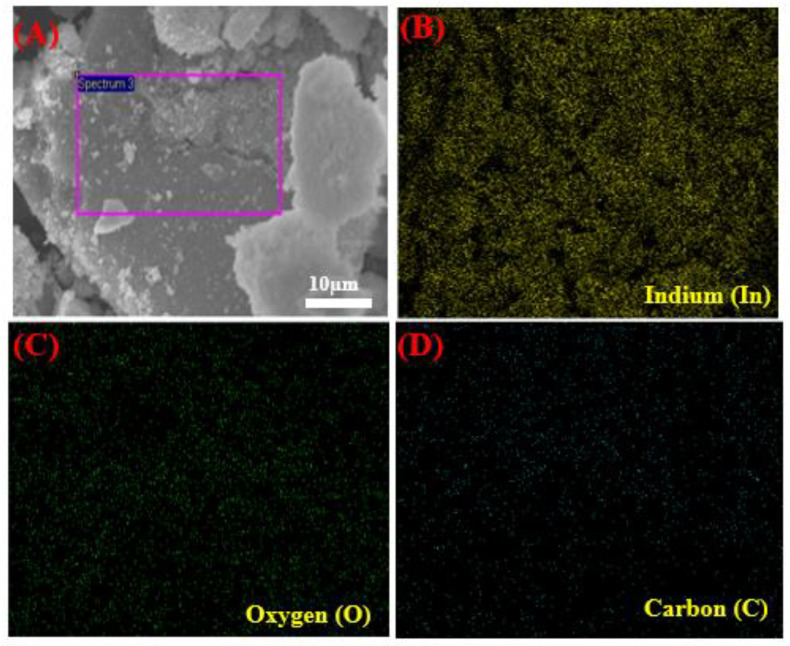
Elemental mapping of In_2_O_3_/RGO NCs: electronic image (**A**), Indium (**B**), Oxygen (**C**), and Carbon (**D**).

Figure 3A–D shows the SEM images with EDX spectra of pure In_2_O_3_ NPs and In_2_O_3_/RGO NCs. As displayed in Figure 3A, the pure In_2_O_3_ NPs were spherically shaped and uniformly distributed. EDX spectra (Figure 3B) shows that the chemical element indium (In) and oxygen(O) were present in pure In_2_O_3_ NPs without any impurities. It can be seen in Figure 3C that the In_2_O_3_ NPs were distributed and attached to the RGO surface. Similarly, the elemental compositions (In, O, and C) in In_2_O_3_/RGO NCs were confirmed in Figure 3D. These percentages are in excellent agreement with the starting precursor. Additionally, Figure 4A–D shows the uniform distribution of the presence of In, O, and C elements in In_2_O_3_/RGO NCs, which further supports the EDX results obtained. All results suggest that the In_2_O_3_/RGO NCs were successfully prepared, which agrees with earlier studies [7,32,41,42].

### 2.3. XPS Analysis

**Figure 5:**
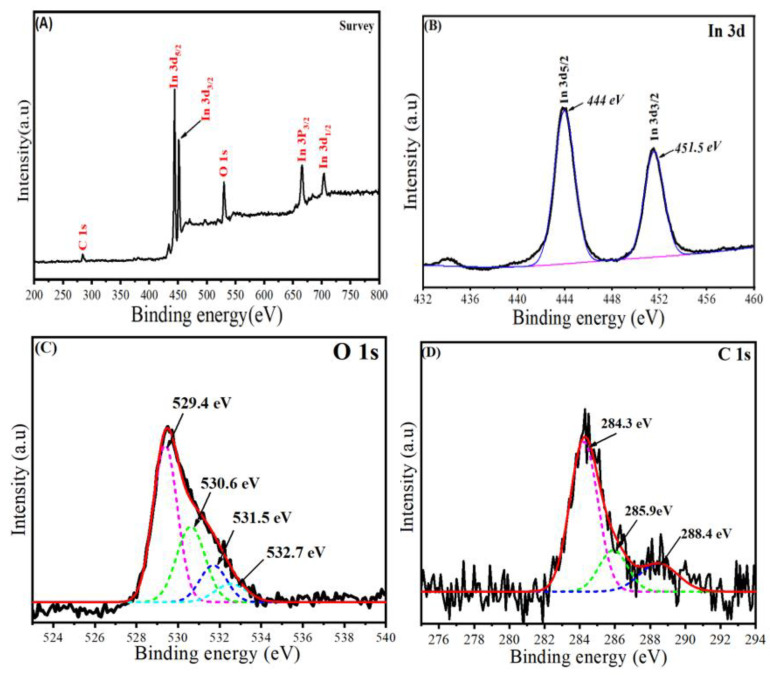
The survey of XPS spectra of In_2_O_3_/RGO NCs (**A**), high-resolution XPS of In 3d (**B**), high-resolution XPS of O 1s (**C**), and high-resolution XPS of C 1s (**D**) in In_2_O_3_/RGO NCs.

Figure 5A–D illustrates the XPS spectra of synthesized In_2_O_3_/RGO NCs. All elemental compositions (In, O, and C) of the In_2_O_3_/RGO NCs were shown in the surveyed XPS spectra (Figure 5A). These results were supported by EDX and mapping results (Figure 3 and Figure 4). Therefore, XPS spectra of In 3d are presented in Figure 5B, which shows the chemical states of indium (In) in the In_2_O_3_/RGO NCs. Hence, the two peaks were observed at 444 and 451.5 eV for In 3d_5/2_ and In3d_3/2_, respectively [36,43]. Earlier studies reported that these peaks are distinctive properties of indium (In) in the +3-oxidation state (In^3+^) within In_2_O_3_ [44]. The multiple peaks of O 1s XPS spectra (Figure 5C) were observed owing to different oxygen species, such as metal oxide (O^2−^) and adsorbed oxygen (O) [45]. The peak centered around 529.4 eV was assigned to the O^2−^ species in In_2_O_3_. Other peaks are located at higher binding energies, around 530–533 eV. These peaks are attributed to the presence of oxygen-containing functional groups on the RGO in the In_2_O_3_/RGO NCs, as reported in previous studies [46,47]. Similarly, the XPS spectra of C 1s (Figure 5D) exhibit three peaks, which are assigned at 284.3, 285.9, and 288.4 eV to different carbon species, graphitic carbon (C=C/C-C), oxygenated carbon (C-O/C=O), and carboxyl groups (O-C=O), respectively [48]. XPS results reveal that the In_2_O_3_/RGO NCs were successfully prepared.

### 2.4. Raman Analysis

**Figure 6:**
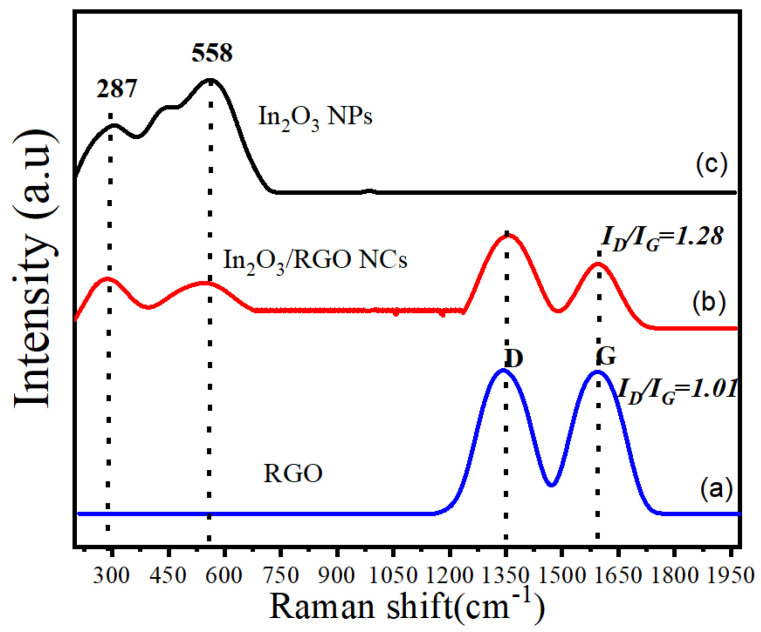
Raman spectra of RGO (**a**), In_2_O_3_/RGO NCs (**b**), and pure In_2_O_3_ NPs (**c**).

Raman spectroscopy is a powerful analytical technique that was applied to investigate the vibrational properties of synthesized samples. Figure 6a–c illustrates the Raman spectra of RGO, pure In_2_O_3_ NPs, and In_2_O_3_/RGO NCs. It can be seen in Figure 6a that the Raman spectra of RGOs have two distinct peaks, which are the D-band (1350 cm^−1^) and G-band (1595 cm^−1^). Moreover, the D-band is associated with the presence of defects or disorders in the graphene structure, while the G-band corresponds to the E_2_g vibrational mode of sp^2^ hybridized carbon atoms [49]. However, the ratio of the intensities of the D-band and G-band (I_D_/I_G_) indicates the degree of disorder in the graphene structure [50]. We observed that the Raman spectra (Figure 6c) of In_2_O_3_ NPs exhibit a sharp and intense peak at around 558 cm^−1^, which corresponds to the Eg mode of In_2_O_3_ [51]. An additional peak in the Raman spectra of In_2_O_3_ NPs was assigned at 287 cm^−1^ due to the E_2_g mode [52]. Hence, the Raman spectra (Figure 6b) of In_2_O_3_/RGO NCs show a combination of the RGO and In_2_O_3_ NPs. In comparison with pure In_2_O_3_ NPs, the D-band and G-band peaks of RGO were still present in the spectra [40,53]. Importantly, these peaks were further associated with Raman spectra of In_2_O_3_ NPs as supported by this study [16]. Obtained results show that the In_2_O_3_/RGO NCs were successfully synthesized due to the strong interaction between the RGO and In_2_O_3_ NPs.

### 2.5. UV Analysis

**Figure 7:**
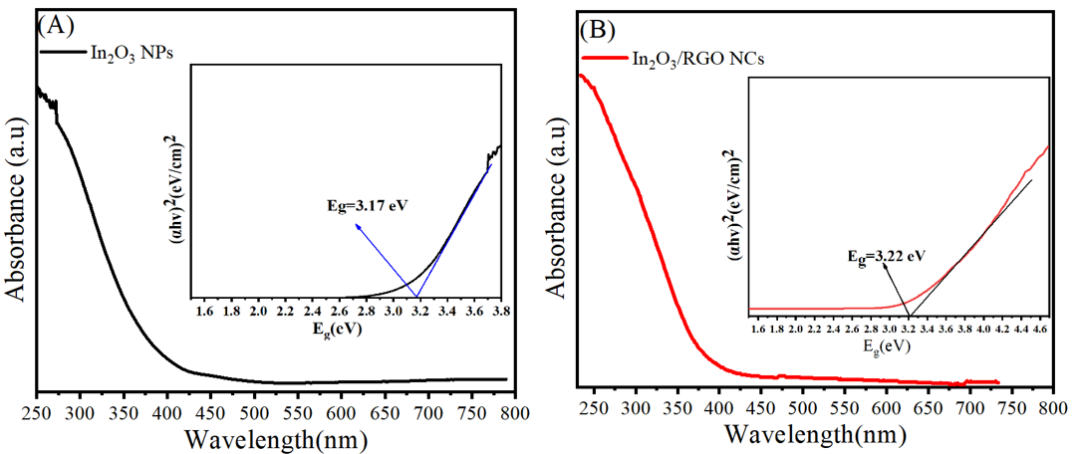
UV–Vis spectra with bandgap energy calculations of pure In_2_O_3_ NPs (**A**) and In_2_O_3_/RGO NPs (**B**).

Investigation of the optical absorption of prepared samples was further performed using UV–Vis spectroscopy. Figure 7A,B depict the peak absorption of pure In_2_O_3_ NPs and In_2_O_3_/RGO NCs. UV–Vis spectra of pure In_2_O_3_ NPs (Figure 7A) exhibit a sharp absorption edge at 392 nm, while the absorption peaks (Figure 7B) of In_2_O_3_ /RGO NCs were also associated at 405 nm. These values indicated that the absorption edge of In_2_O_3_ /RGO NCs is shifting to a higher wavelength compared to pure In_2_O_3_ NPs. Thus, the phenomena shows a reduction in the bandgap energy of the In_2_O_3_ /RGO due to the presence of RGO in pure In_2_O_3_ NPs as reported by these investigators [32,54]. As depicted in Table 1, the band gap energies of In_2_O_3_ NPs and In_2_O_3_/RGO NPs were 11.17 eV and 3.22 eV, respectively. Owing to the strong interaction between pure In_2_O_3_ and RGO nanosheet, the band gap energy increased with the dopant of RGO. UV–Vis results indicated that RGO doping plays a role in improving the optical properties of In_2_O_3_ for photocatalytic and biomedicine applications.

### 2.6. PL Analysis

**Figure 8:**
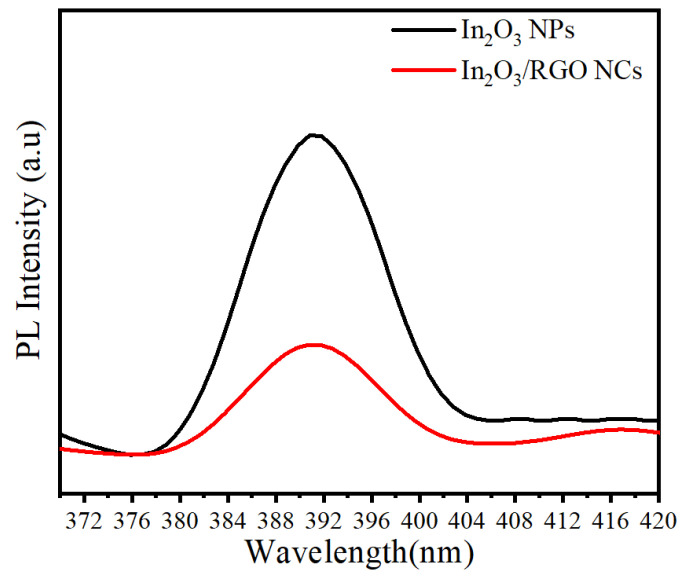
PL spectra of pure In_2_O_3_ NPs and In_2_O_3_/RGO NCs.

Photoluminescence (PL) analysis was employed to ascertain the segregation of photo-generated electrons and holes within the synthesized samples. Figure 8 presents the room-temperature photoluminescence (PL) spectra of pure In_2_O_3_ NPs and In_2_O_3_/RGO NCs. Excitation was achieved using a 320 nm wavelength. The PL spectra show an emission peak at 391.11 nm for all the prepared NPs as reported in a previous study [55]. After the dopant of the RGO sheet, the emission peak of In_2_O_33_/RGO NCs was highly decreased compared with pure In_2_O_3_ NPs. It was noticed that the PL intensity of the In_2_O_3_/RGO NCs (indicated by the red line) was lower compared to pure In_2_O_3_ NPs. This could be attributed to enhanced charge separation, lifespan of the electron-hole pair, and charge efficiency [56]. PL results indicate that the prepared In_2_O_3_/RGO NCs can be applied to enhance photocatalytic activity and biomedical applications.

### 2.7. Photocatalytic Performance

**Figure 9:**
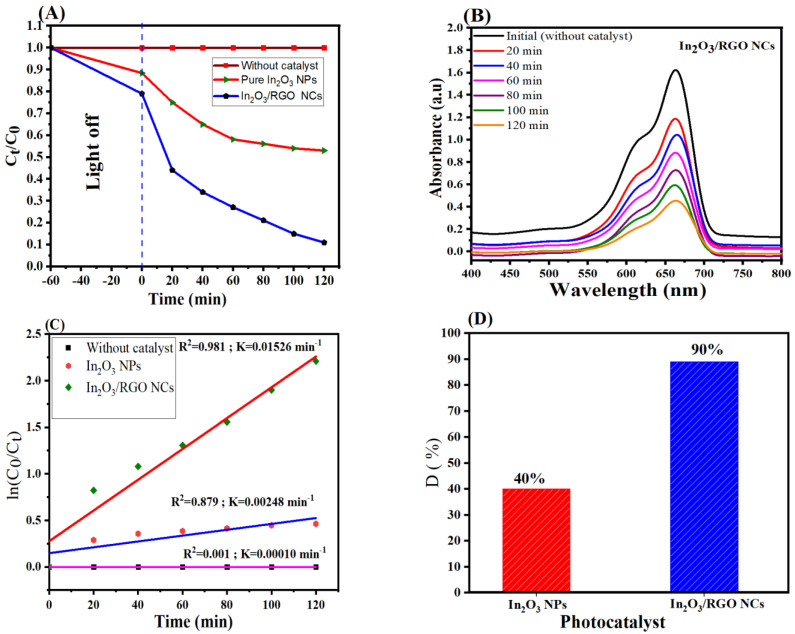
Photocatalytic degradation of MB dye by pure In_2_O_3_ NPs and In_2_O_3_ /RGO NCs under UV light irradiation (**A**), UV–Vis spectra of MB in aqueous solution (**B**), linear relationship between ln(*C*_*t*_/*C*_0_) and irradiation time (min) (**C**), and Degradation efficiency of MB of pure In_2_O_3_ NPs and In_2_O_3_ /RGO NCs (**D**).

The photocatalytic performance of synthesized samples was assessed by the degradation of MB dye under UV light irradiation. Figure 9A–D shows the results of the photocatalytic pure In_2_O_3_ NPs and In_2_O_3_/RGO NCs under UV light irradiation by degradation of MB dye. As shown in Figure 9A, the In_2_O_3_/RGO NCs exhibited significantly higher photocatalytic activity than pure In_2_O_3_ NPs. Furthermore, the absorption peak (Figure 9B) of MB at 664 nm was gradually decreased through the degradation time (120 min) of In_2_O_3_/RGO NCs. The linear relationship between the natural logarithm of the ratio of the initial (ln(*C*_0_/*C*_*t*_)) and the degradation time (min) is shown in Figure 9C. The high linearity of the plot indicates that the photocatalytic degradation of MB of In_2_O_3_/RGO NCs follows a first-order kinetics reaction. However, the rate constant (K) values of pure In_2_O_3_ NPs and In_2_O_3_/RGO NCs were 0.00248 min^−1^ and 0.01526 min^−1^, respectively [32]. It can be seen in Figure 9D that the degradation efficiencies of the MB solution of pure In_2_O_3_ NPs and In_2_O_3_/RGO NCs under the same conditions were 40% and 90 % after 120 min, respectively. Results showed that the In_2_O_3_/RGO NCs have superior photocatalytic activity towards the degradation of MB under UV light irradiation compared to pure In_2_O_3_ NPs. This enhanced photocatalytic activity of In_2_O_3_/RGO NCs could be attributed to increasing the surface area and enhancing the light absorption capability of the NCs by RGO doping into the In_2_O_3_ NPs [57]. A comparison of In_2_O_3_/RGO NCs with several studies in the degradation of methylene blue (MB) dye is shown in Table 2. All results suggest that the In_2_O_3_/RGO NCs can be applied to wastewater and cancer treatment.

### 2.8. Potential Photocatalytic Mechanism

**Figure 10:**
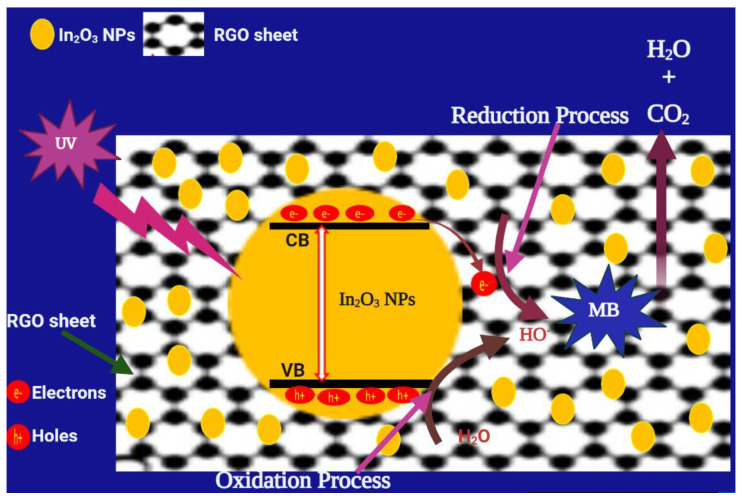
The possible mechanism of MB degradation of In_2_O_3_/RGO NCs under UV light irradiation.

Figure 10 interprets the photocatalytic mechanism used by In_2_O_3_/RGO NCs for the degradation of MB dye. Particularly, the mechanism begins with the absorption of UV light by In_2_O_3_/RGO NCs, which promotes the generation of electron(e^−^)-hole (h^+^) pairs. The excited electrons in the valance band (VB) move to the conduction band (CB) which produces holes (h^+^) in the VB. These excited electrons are transferred from the conduction band (CB) of In_2_O_3_ to the surface of the RGO sheet. Furthermore, these electrons reacted with oxygen and water to produce hydroxyl radicals (•OH) and oxygen radicals. Then, these radicals degraded MB to produce intermediate products (H_2_O and CO_2_).

### 2.9. Cytotoxicity and Compatibility Investigations

**Figure 11:**
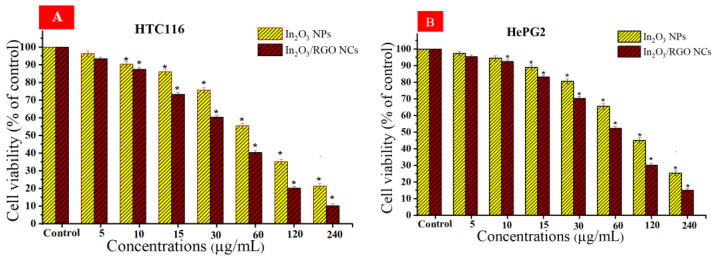

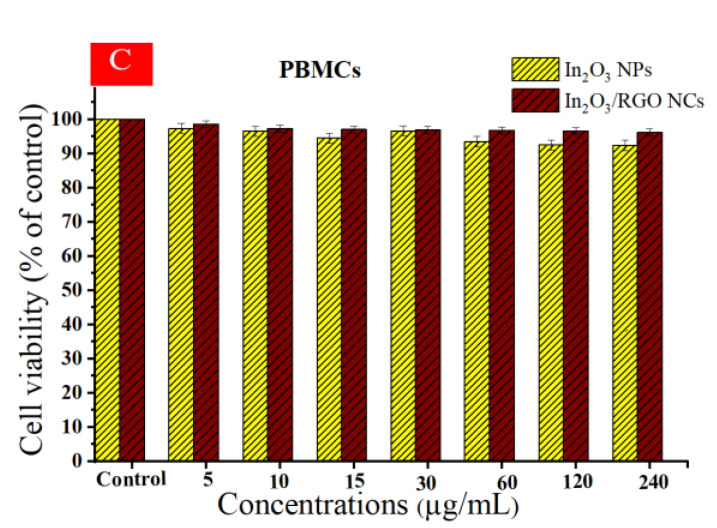
Cell viability after 24 h of incubation with prepared samples at concentrations (5–240 μg/mL), HC116 cell line (**A**), HepG2 cells (**B**), and the cytocompatibility of prepared samples in human peripheral blood mononuclear cells (PBMCs) by using the MTT assay (**C**). * Significantly different from control (*p* < 0.05).

Different studies have examined the anticancer potential of graphene nanocomposites (NCs) based on metal oxides against various types of cancer cells [62,63,64]. Cytotoxicity and cytocompatibility of prepared samples at different concentrations against (HC116 and HepG2) as well as in normal PBMCs were illustrated in Figure 11A–C. As depicted in Figure 11A, the cell viability of the HC116 cell lines decreased with increasing concentrations of the prepared samples. Similarly, the cytotoxicity effects of both the prepared samples against the HepG2 cell line were increased with increasing concentrations. These Results show that the In_2_O_3_/RGO NCs have higher cytotoxicity toward HC116 and HepG2 cancer cells in comparison with pure In_2_O_3_ NPs. Significantly, Figure 11C shows the cytocompatibility of the prepared samples against normal human PBMCs. It is observed that the percentage of cell viability remained relatively constant across all concentrations of the prepared samples. The collected results suggest that the addition of RGO to pure In_2_O_3_ NPs enhances anticancer efficacy. IC_50_ values of synthesized samples are presented in Table 3.

## 3. Experimental Section

### 3.1. Materials and Cells

Indium nitrate hydrate (In(NO_3_)_3_. xH_2_O) (99.99% trace metals basis), reduced graphene oxide (RGO), ethylene glycol (EG)(anhydrous,99.8%), and sodium hydroxide (NaOH) (reagent grade,98%), methylene blue (MB) dye (≥97.0% (calc. based on dry substance, AT)) were purchased from Sigma-Aldrich (Millipore-Sigma, St Louis, MO, USA). Human colorectal (HCT116) and liver (HepG2) cancer cells were purchased from American Type Culture Collection (ATTC, Manassas, WV, USA). Additionally, human normal peripheral blood mononuclear cells (PBMCs) were obtained from the microbiological Lab at KSU as shown in this study [65]. All chemicals were used as received without further purification. Ethanol (EtOH, 99.9%) and deionized water (DI water) were used as solvents.

### 3.2. Synthesis of Pure In_2_O_3_ NPs and In_2_O_3_/RGO NCs

**Scheme 1:**
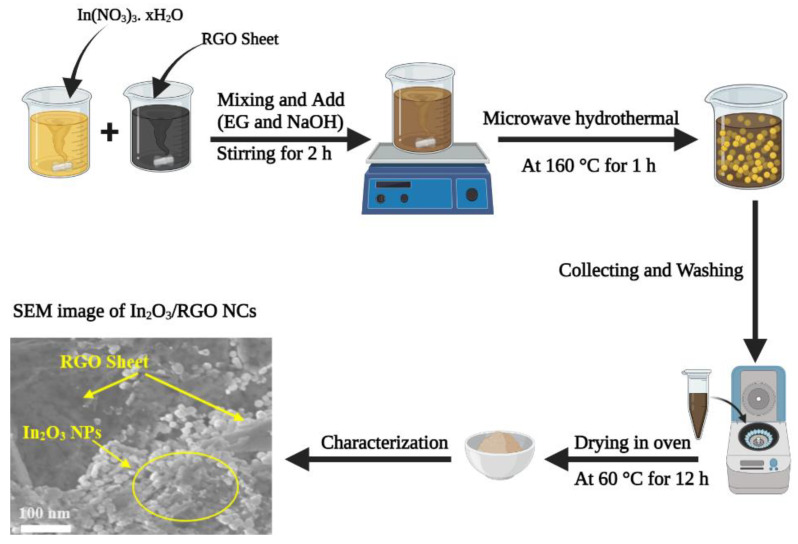
Synthesis protocol for preparation of In_2_O_3_/RGO NCs.

The preparation of pure In_2_O_3_ NPs and In_2_O_3_/RGO NCs were carried out via the microwave hydrothermal method [24]. A sample of 50 mg of RGO nanosheet was dissolved in 20 mL of deionized water with magnetic stirring to get an homogeneous GO suspension as the first solution. Similarly, the 0.5 g of In (NO_3_)_3_. xH_2_O was also dispersed in 40 mL of deionized water with magnetic stirring as a second solution. Subsequently, the first and second solutions were mixed with magnetic stirring. Then, NaOH (1 M) and EG (5 mL) were added to the mixture under vigorous stirring for 2 h. The mixture solution was transferred to a Teflon-lined stainless-steel autoclave (100 mL). After that, the autoclave was sealed and subjected to microwave treatment in a microwave oven (2450 MHz) at 160 °C for 1 h. After the microwave hydrothermal reaction, the vessel was removed from the microwave reactor. After cooling to room temperature, the obtained precipitates were collected by centrifugation, washed with deionized water and ethanol, and dried at 60 °C for 12 h. The pure In_2_O_3_ NPs were further synthesized by the same method but without adding RGO. The synthesis protocol for In_2_O_3_/RGO nanocomposites is depicted in Scheme 1.

### 3.3. Characterization

Measurements of the crystallinity and purity and crystal size of synthesized samples were carried out using X-ray diffractometer (XRD) analysis (PanAnalytic X’Pert Pro, Malvern Instruments, Malvern, UK). Transmission electron microscopy (TEM) (200 kV,2100F, JEOL, Inc., Tokyo, Japan) and scanning electron microscopy (SEM) (SEM, JSM-7600F, JEOL, Inc. Tokyo, Japan) (JSM-7600F, JEOL, Inc.) were further employed to assess the morphologies and the distribution of prepared samples. Energy dispersive X-ray spectroscopy (EDX) (SEM, JSM-7600F, JEOL, Inc. Tokyo, Japan) (JSM-7600F, JEOL, Inc.) was also used to confirm the presence of elements in synthesized samples. Furthermore, the chemical state and elemental composition of obtained samples were determined by X-ray photoelectron spectroscopy (XPS) (PHI-5300 ESCA PerkinElmer, Boston, MA, USA). Additionally, Raman spectroscopy was used to study the vibrational modes of the synthesized samples. The optical properties of synthesized samples were assayed using UV–Vis (Hitachi U-2600) and Photoluminescence (PL) (Hitachi F-4600) spectroscopy.

### 3.4. Photocatalytic Experiment

The degradation of MB dye solution under UV light irradiation (300 W xenon lamp) was carefully used to evaluate the photocatalytic properties of prepared samples. The 20 mg of pure In_2_O_3_ NPs and 10 mg/L of MB dye solution were added to a 250 mL beaker of deionized water. Then, the solution suspension was further stirred in the dark for 30 min to achieve an adsorption–desorption equilibrium between the samples and the MB solution. Subsequently, this solution was irradiated under UV light irradiation with magnetic stirring. At 20 min intervals, 3 mL of the solution suspension was taken during the photocatalytic reaction. Then, the concentration of the photodegraded MB solution was further measured by a UV–Visible spectrophotometer after removing the photocatalyst of this solution. The photocatalytic actvity of the In_2_O_3_/RGO NCs was also evaluated under the same experimental conditions. The degradation efficiency of MB dye was calculated using the following formula of $D\;\left(\%\right)=\left[\left(C_{0}-C_{t}\right)/C_{t}\right]\;\times \;100$. Where *C_0_* represents the dye concentration at the beginning, while *C_t_* refers to the dye concentration after a certain time ‘t’ (measured in min). To test the stability, the catalysts were centrifuged, washed with deionized water and ethanol, and dried at 60 °C for 12 h. Hence, centrifuged catalysts were used several times after drying at 70 °C for 10 h.

### 3.5. Cytotoxicity Assessment

The cytotoxicity of synthesized In_2_O_3_ NPs and In_2_O_3_/RGO NCs was further assessed using human colorectal (HCT116) and liver (HepG2) cancer cells by using the MTT assay. Importantly, the biocompatibility of prepared samples was tested agonist human normal peripheral blood mononuclear cells (PBMCs). Cells were cultured in Dulbecco’s Modified Eagle’s Medium (DMEM) containing 10% fetal bovine serum (FBS) and 1% penicillin-streptomycin at 37 °C in a humidified atmosphere of 5% CO_2_. Thus, cells were seeded in a 96-well plate at a density of 5 × 10^3^ cells/well and incubated for 24 h. Then, the cells were treated with various concentrations (5–240 μg/mL) of pure In_2_O_3_ NPs and In_2_O_3_/RGO NCs with incubation for 24 h. The cell viability was measured using absorbance at 570 nm using a microplate reader.

### 3.6. Statistical Analysis

The statistical analysis was performed using a one-way analysis of variance (ANOVA) followed by the multiple comparison test of Tukey. A value of *p* < 0.05 was considered statistically significant.

## 4. Conclusions

In the present study, we used a single-step microwave hydrothermal method for the fabrication of In_2_O_3_ on the surface of RGO. The prepared samples have been analyzed using XRD, TEM, SEM, EDX, XPS, Raman spectroscopy, UV–Vis, and PL spectroscopy. XRD data show that the synthesized samples exhibited high crystallinity with reduced crystal size after RGO addition. TEM and SEM images showed that In_2_O_3_ NPs were spherically shaped and uniformly embedded on RGO sheets. EDX and XPS analysis confirmed the elemental composition without impurities. The PL study suggests a hindrance in the recombination rate of electron-holes of In_2_O_3_/RGO NCs in comparison to pure In_2_O_3_ NPs. Raman analysis further confirmed the successful fabrication of In_2_O_3_ on the RGO surface. In the degradation experiment, it was observed that the In_2_O_3_/RGO NCs display better photocatalytic performance as compared to pure In_2_O_3_NPs. The biological data revealed that In_2_O_3_/RGO NCs have higher anticancer activity in human colorectal (HCT116) and liver (HepG2) cancer cells than those of In_2_O_3_NPs. Moreover, In_2_O_3_/RGO NCs demonstrated higher cytocompatibility with human normal peripheral blood mononuclear cells (PBMCs) compared to pure In_2_O_3_ NPs. These results indicate that incorporating RGO enhances the photocatalytic and anticancer properties of In_2_O_3_ NPs. This research emphasizes the promising capabilities of In_2_O_3_/RGO NCs as highly effective photocatalysts and therapeutic materials.

## Figures and Tables

**Table 1:** Comprehensive Characterization of the Structural and Optical Properties of In_2_O_3_NPs and In_2_O_3_/RGO NCs.

Prepared Samples	XRD Size (nm)	TEM Size (nm)	Bandgap Energy (Eg)
In_2_O_3_ NPs	17	12	3.17
In_2_O_3_/RGO NCs	13	10	3.22

**Table 2:** Comparison of degradation of methylene blue (MB) dye using In_2_O_3_/RGO NCs and previously reported samples.

Catalyst Used	Concentration of MB/Amount of Catalyst	Light Source	Exposure Time (min)	D (%)	References
In_2_O_3_/RGO NCs	10 ppm/20mg	UV light	120 min	90%	Present work
Zn-In_2_O_3_ NPs	20 ppm/50 mg	Visible-light	180 min	94.1%	[58]
C-In_2_O_3_/BiOI NCs	10 ppm/25 mg	Visible-light	90min	99%	[59]
In_2_O_3_/SnO_2_ NCs	4 ppm/0.2 mg	UV light	180 min	50.9%	[60]
ZnO/rGO NCs	10ppm/20 mg	Visible-light	120 min	99.1%	[61]
In_2_O_3_-ZnO NCs	20 ppm/20 mg	UV light	60 min	95.3%	[44]
Ag-In_2_O_3_ NPs	10 ppm/30 mg	UV light	360 min	92%	[42]

**Table 3:** IC_50_ Values of Synthesized Compounds in HC116 and HepG2Cancer Cell Lines IC_50_.

Samples	Cancer Cells Lines	
	HepG2 cells	HCT116 cells
In_2_O_3_ NPs	70.47	98.69
In_2_O_3_/RGO NCs	40.31	57.96

## Data Availability

Not applicable.

## Sample Availability

Samples of the compounds in this study are available from the authors.
